# Members of the Pmp protein family of *Chlamydia pneumoniae* mediate adhesion to human cells via short repetitive peptide motifs

**DOI:** 10.1111/j.1365-2958.2010.07386.x

**Published:** 2010-10-06

**Authors:** Katja Mölleken, Eleni Schmidt, Johannes H Hegemann

**Affiliations:** Institut für Funktionelle Genomforschung der Mikroorganismen, Heinrich-Heine-Universität Düsseldorf, Universitätsstr. 1Gebäude 25.02.U1, 40225 Düsseldorf, Germany

## Abstract

*Chlamydiae* sp. are obligate intracellular pathogens that cause a variety of diseases in humans. Adhesion of the infectious elementary body to the eukaryotic host cell is a pivotal step in chlamydial pathogenesis. Here we describe the characterization of members of the polymorphic membrane protein family (Pmp), the largest protein family (with up to 21 members) unique to *Chlamydiaceae*. We show that yeast cells displaying Pmp6, Pmp20 or Pmp21 on their surfaces, or beads coated with the recombinant proteins, adhere to human epithelial cells. A hallmark of the Pmp protein family is the presence of multiple repeats of the tetrapeptide motifs FxxN and GGA(I, L, V) and deletion analysis shows that at least two copies of these motifs are needed for adhesion. Importantly, pre-treatment of human cells with recombinant Pmp6, Pmp20 or Pmp21 protein reduces infectivity upon subsequent challenge with *Chlamydia pneumoniae* and correlates with diminished attachment of *Chlamydiae* to target cells. Antibodies specific for Pmp21 can neutralize infection *in vitro*. Finally, a combination of two different Pmp proteins in infection blockage experiments shows additive effects, possibly suggesting similar functions. Our findings imply that Pmp6, Pmp20 and Pmp21 act as adhesins, are vital during infection and thus represent promising vaccine candidates.

## Introduction

*Chlamydiae* are Gram-negative bacteria with compact genomes, and some species represent significant threats to human health. *Chlamydia trachomatis* is the most prevalent sexually transmitted bacterial pathogen worldwide (Schachter, 1999). *Chlamydia pneumoniae* is an important respiratory pathogen, causing pneumonia and bronchitis, and is associated with several chronic diseases (Schachter, 1999). Despite the clinical relevance of *Chlamydia*, no vaccine is available for use in humans (Brunham and Rey-Ladino, 2005; Puolakkainen, 2009). *Chlamydia* species have a unique biphasic developmental cycle, alternating between the infectious elementary body (EB) and the metabolically active, intracellular reticulate body (RB) that replicates in eukaryotic cells (for reviews see Moulder, 1991; Hatch, 1999). The mechanisms underlying EB infectivity are unclear (Campbell and Kuo, 2006; Scidmore, 2006).

Polymorphic membrane proteins (Pmps) found in species of the *Chlamydiaceae* are candidate adhesins (Longbottom *et al*., 1998; Stephens *et al*., 1998; Grimwood and Stephens, 1999; Kalman *et al*., 1999; Knudsen *et al*., 1999; Read *et al*., 2003; Wehrl *et al*., 2004; Crane *et al*., 2006). The *C. trachomatis pmp* gene family has nine members (Stephens *et al*., 1998; Grimwood and Stephens, 1999; Tan *et al*., 2006), representing six subtypes: *A*, *B/C*, *D*, *E*, *G* and *H*. *C. pneumoniae* has 21 *pmp* genes, 13 of which (*pmp1*–*pmp13*) belong to subtype *G* (Grimwood and Stephens, 1999; Kalman *et al*., 1999). The *pmp* genes make up more than 5% of the total coding capacity of the genome, and are unique to the *Chlamydiaceae*, suggesting that their products might be virulence factors (Horn *et al*., 2004). The various Pmp families show little similarity in amino acid sequence, but all members contain multiple repeats of the motifs GGA(I, L, V) and FxxN (Grimwood and Stephens, 1999; Rockey *et al*., 2000). The GGAI sequence is predicted to be present in only 10 other proteins (and only in one copy per protein) in the chlamydial proteomes, underscoring the over-representation of the motif in the Pmp families (Grimwood and Stephens, 1999). Similarly, the FxxN motif is found on average 13.6 times per Pmp protein in *C. trachomatis* and 11.3 times in *C. pneumoniae*, respectively, while its average incidence in the rest of the proteome is 0.73 (*C. trachomatis*) or 0.84 (*C. pneumoniae*) (Grimwood and Stephens, 1999).

**Fig. 1:**
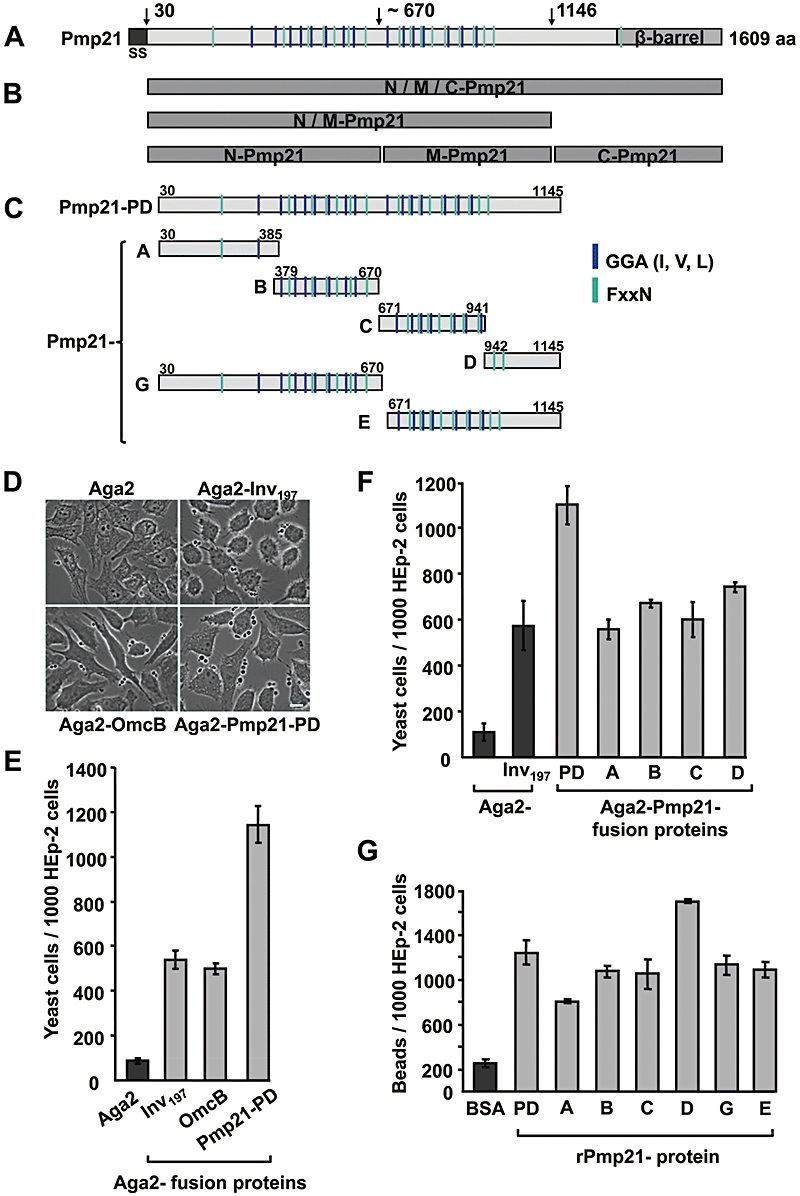
Pmp21 mediates adherence to human cells.A. Schematic representation of Pmp21. The full-length protein is depicted at the top, with the N-terminal signal sequence (SS) and the C-terminal β-barrel domain (β-barrel). Each of the tetrapeptide motifs GGA(I, L, V) and FxxN in the central passenger domain is marked. Two known proteolytic cleavage sites in Pmp21 are indicated by arrows (Vandahl *et al*., 2002; Wehrl *et al*., 2004).B. Processed forms of Pmp21 detected by proteome analysis (Grimwood *et al*., 2001; Vandahl *et al*., 2002; Wehrl *et al*., 2004).C. The Pmp21 passenger domain (Pmp21-PD) and its subdomains Pmp21-A to -G studied in this work.D. Micrographs of HEp-2 cells incubated with yeast cells expressing the indicated proteins. Bar 10 µm (see also Fig. S1).E. Binding of yeast cells expressing Aga2, Aga2–Inv_197_, Aga2–OmcB and Aga2–Pmp21-PD to HEp-2 cells (see also Fig. S2).F. Binding of yeast cells expressing Aga2, Aga2–Inv_197_, Aga2–Pmp21-PD, Aga2–Pmp21-A, -B, -C and -D to HEp-2 cells.G. Adhesion of latex beads coated with the indicated proteins to HEp-2 cells. Beads (1 × 10^6^) coated with 100 µg BSA, rPmp21-PD, rPmp21-A, -B, -C, -D, -E and -G were incubated with 1 × 10^5^ HEp-2 cells and the number of beads associated with HEp-2 cells was determined by microscopy.The data in (E)–(G) are based on the inspection of 1000 HEp-2 cells per experiment, and the results for each construct are derived from four independent experiments. Error bars indicate standard deviations.

Pmp proteins resemble classical autotransporters (ATs), with (i) an N-terminal sequence for *sec*-dependent translocation across of the cytoplasmic membrane, (ii) a C-terminal β-barrel domain with a phenylalanine at the end suggestive for a outer membrane localization of Pmp proteins, and (iii) a central passenger domain (PD) responsible for the protein's function that can remain surface-localized or be secreted (e.g. Pmp21 in Fig. 1A) (Henderson and Lam, 2001; Dautin and Bernstein, 2007; Wells *et al*., 2007). The Pmp PDs, which carry nearly exclusively the tetrapeptide repeats, probably adopt a parallel β-helix structure (Vandahl *et al*., 2002). Like other AT proteins, Pmps apparently undergo proteolytic processing (e.g. Pmp21 in Fig. 1B) (Vandahl *et al*., 2001; 2002; Vretou *et al*., 2003; Wehrl *et al*., 2004; Kiselev *et al*., 2009; Swanson *et al*., 2009). However, there is no evidence for secretion of Pmps by the AT pathway.

Some Pmps are known to be surface-located on infectious EBs, and are thus positioned at the interface between pathogen and the infected cell (Vandahl *et al*., 2002; Wehrl *et al*., 2004; Crane *et al*., 2006; Kiselev *et al*., 2009; Swanson *et al*., 2009). Antibodies against Pmp21 from *C. pneumoniae* or PmpD from *C. trachomatis* inhibit infection *in vitro* (Wehrl *et al*., 2004; Crane *et al*., 2006). Patients infected with either species show significant serological responses to various Pmps (Bunk *et al*., 2008; Tan *et al*., 2009). The high degree of variation found among *pmp* gene families suggests that they are under selective pressure (Grimwood and Stephens, 1999; Shirai *et al*., 2000; Pedersen *et al*., 2001; Rocha *et al*., 2002; Gomes *et al*., 2005; 2006; Tan *et al*., 2009). Indeed, *C. trachomatis* inclusions formed in cultured cells exhibit variability in the sets of Pmps they express, suggesting they confer antigenic diversity (Tan *et al*., 2010).

We show that Pmp6, Pmp20 and Pmp21, representing three of the six subtypes (B/C, D, G), act as adhesins during infection by *C. pneumoniae*. FxxN and GGA(I, L, V) motifs are necessary for this activity. Pmp21 has several adhesive domains, but the strongest cell-binding and infection-blocking activities are associated with processed forms of the protein known to occur *in vivo*. A 32-residue Pmp21 peptide including a FxxN/GGAV doublet can competitively reduce a *C. pneumoniae* infection. Interestingly, a combination of two different Pmp proteins in infection blockage experiments shows additive effects, possibly suggesting similar functions.

## Results

### *C. pneumoniae* Pmp21 mediates adhesion to human epithelial cells

Pmp21 and PmpD from *C. trachomatis* are targets of neutralizing antibodies and may function in pathogenesis (Wehrl *et al*., 2004; Crane *et al*., 2006). To ask if Pmp21 is an adhesin, we used a yeast display system (Fig. S1) (Moelleken and Hegemann, 2008). The PD of Pmp21 (residues 30–1144) (Fig. 1C) was fused to the yeast surface protein Aga2p (Aga2–Pmp21-PD) and the cells were tested for adhesion to human epithelial HEp-2 cells. Yeast cells displaying Aga2–Pmp21-PD, but not Aga2 alone or Aga2 fused to the chlamydial outer membrane protein OmpA (MOMP), adhered to HEp-2 cells (Fig. 1D; Fig. S2). Indeed, cells displaying Pmp21-PD showed significantly stronger adhesion to HEp-2 cells than did cells presenting *Yersinia pseudotuberculosis* invasin (Aga2–Inv_197_) or chlamydial OmcB (Fig. 1E) (Dersch and Isberg, 1999; Moelleken and Hegemann, 2008). Thus, Pmp21-PD can mediate adhesion to human cells.

### Several domains of Pmp21 can mediate adhesion to HEp-2 cells

To identify the parts of *C. pneumoniae* Pmp21 that mediate binding, we tested four subdomains of Pmp21-PD in the yeast system (Fig. 1C). Any one of them fused to Aga2 mediated adherence to HEp-2 cells, although all were less effective than Pmp21-PD (Fig. 1F).

We also performed latex bead assays with Pmp21 (Dersch and Isberg, 1999). We purified Pmp21-PD and the four Pmp21 domains A–D, expressed as His-tagged proteins in *Escherichia coli*, and incubated them with latex beads. The coated beads were then tested for adhesion to HEp-2 cells. Beads loaded with recombinant MBP or MBP-OmpA or BSA exhibited scarcely any binding to HEp-2 cells, while beads bearing His- or MBP-tagged invasin or Pmp21-PD (or any of its four fragments) clearly bound to human cells (Fig. 1G; Fig. S2). Thus, Pmp21-PD has several independent binding domains that mediate adherence to human cells. Published work suggests that Pmp21 is cleaved *in vivo*, and infectious EBs probably carry at least three different processed forms (N-Pmp21, M-Pmp21 and C-Pmp21) (Fig. 1B). The last, comprising the β-barrel unit, probably remains in the outer membrane (Grimwood *et al*., 2001; Vandahl *et al*., 2002; Wehrl *et al*., 2004). We created two variants, Pmp21-E and Pmp21-G, corresponding to N-Pmp21 and M-Pmp21 respectively (Fig. 1C). Latex beads coated with either adhered to HEp-2 cells just as well as the other Pmp21 variants tested (Fig. 1G). Thus, the processed N-Pmp21 and M-Pmp21 forms found on EBs can bind to human cells.

### Adhesion to human cells requires at least two tetrapeptide motifs

**Fig. 2:**
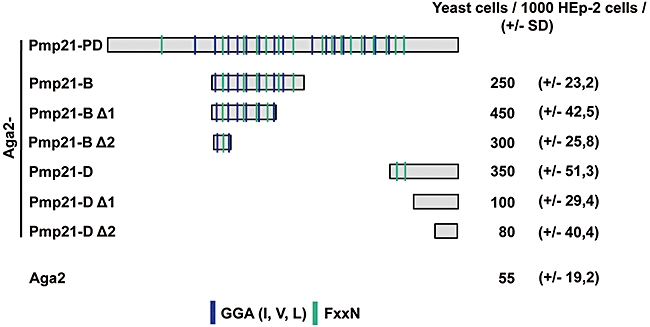
Identification of minimal Pmp21 adhesion domains. Schematic representation of the deletion variants generated in the Pmp21-B and Pmp21-D domains, and summary of their relative adhesion activities when expressed as Aga2 fusions on yeast cells. The position of each of the tetrapeptide motifs GGA(I, L, V) and FxxN is marked. The relative binding affinity of yeast cells expressing each Pmp21 deletion variant for HEp-2 cells is indicated on the right, and is expressed as the number of yeast cells bearing the indicated constructs found adhering to 1000 HEp-2 cells. Adhesion was quantified as explained in the legend to Fig. 1. SD, standard deviation.

The only feature common to the variants tested so far is the presence of repeated GGA(I, L, V) and FxxN motifs (Fig. 1C). We selected Pmp21-B and Pmp21-D, which showed comparable adhesion properties in the yeast assay, but have very different numbers of tetrapeptide repeats (Fig. 2), for further study. Deletion experiments revealed that the 50-residue Pmp21-BΔ2, with one FxxN and two GGA(I, V, L) repeats, behaved like Pmp21-B. In contrast, N-terminal truncation of Pmp21-D, deleting the only two tetrapeptide FxxN motifs present, resulted in a significant reduction (Pmp21-DΔ1) or complete loss (Pmp21-DΔ2) of adhesiveness (Fig. 2). Thus Pmp21 domains with two FxxN motifs (Pmp21-D) and one FxxN and two GGA(I, V, L) motifs (Pmp21-BΔ2) both show significant adhesion to HEp-2 cells.

**Fig. 3:**
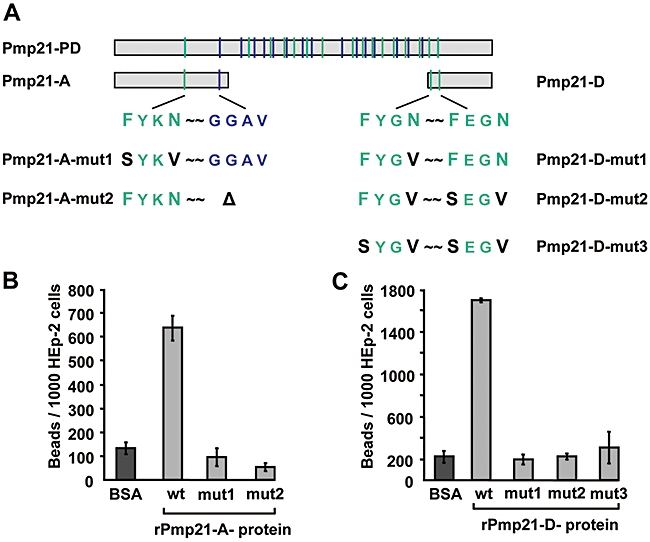
Mutational analysis of Pmp21 adhesion domains.A. Schematic representation of the alterations introduced into Pmp21-A and Pmp21-D (see Fig. 1C). The position and specific sequence of each of the tetrapeptide motifs GGA(I, L, V) and FxxN are depicted.B and C. Binding of latex beads (1 × 10^6^) coated with the indicated recombinant Pmp21 variants to 1 × 10^5^ HEp-2 cells. For details, see the legend to Fig. 1.

To confirm that these motifs are necessary for Pmp21-mediated adhesion to human cells, we chose Pmp21-A and Pmp21-D (Fig. 3A). We mutated the FYKN motif in Pmp21-A to SYKV, leaving the GGAV motif unchanged. Beads bearing Pmp21-A-mut1 exhibited no adhesion to human cells. Similarly, deletion of the GGAV motif in Pmp21-A, leaving the FYKN motif intact, abrogated adhesion (Pmp21-A-mut2, Fig. 3A and B). In Pmp21-D we changed a single residue in the N-terminal FYGN motif, keeping the FEGN motif intact, or changed both motifs. All three mutant forms of Pmp21-D showed no adhesive capacity (Fig. 3A and C). Hence the FxxN and GGAV motifs are necessary for binding of the Pmp21A and Pmp21D domains to HEp-2 cells. A minimum of two copies of either motif (FxxN + GGAV or FxxN + FxxN) is required for adhesion.

### *C. pneumoniae* Pmp21 is located on the surface of EBs and RBs during infection

**Fig. 4:**
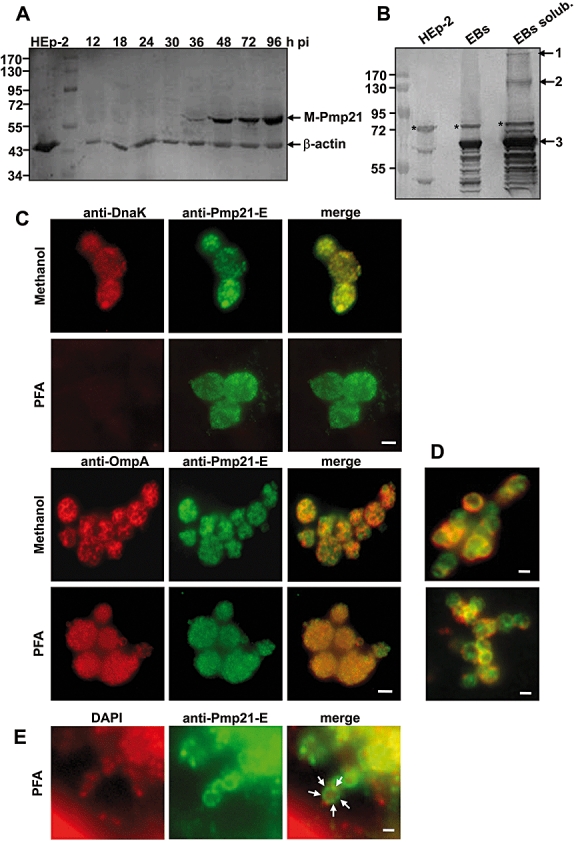
*C. pneumoniae* Pmp21 is expressed on the bacterial surface during infection.A. Pmp21 expression was assessed by immunoblot analysis with anti-Pmp21-D using lysates from HEp-2 cells infected with *C. pneumoniae* GiD at moi 1 for 12–96 h. The host marker actin was used as loading control.B. Immunoblot analysis of total protein lysates prepared from gradient-purified EBs. EBs (∼10^7^) were resuspended in PBS or in PBS containing 2% SDS, 2% sarcosyl and 10 mM DTT for 30 min at 37°C, and analysed by SDS-PAGE using anti-Pmp21-D serum (antigen-purified). Band 1 corresponds in size to N/M/C-Pmp21, band 2 to the processing intermediate N/M-Pmp21 and band 3 to the fully processed M-Pmp21 form. A star marks an unspecific cross-reacting band seen in uninfected HEp-2 cells and in EBs purified from infected HEp-2 cells.C. Localization of Pmp21 during infection. HEp-2 cells were infected with *C. pneumoniae* for 48 h, and fixed with methanol (methanol) for visualization of intra- and extrachlamydial antigens. Antibodies directed against DnaK, OmpA and Pmp21 detected chlamydial particles in the inclusion. Fixation with formaldehyde (PFA) and permeabilization with 0.5% Triton X-100 allowed visualization of extrachlamydial antigens (Birkelund *et al*., 1990). Bar 5 µm.D. Higher magnification of inclusions seen in methanol-fixed cells 48 h post infection with *C. pneumoniae*. Antibodies directed against OmpA (red) and Pmp21 (green) detect chlamydial particles in the inclusion. Note the ring-shaped patterns often seen upon labelling of OmpA and Pmp21. Bar 1 µm.E. Higher magnification of inclusions seen in formaldehyde/Triton X-100-fixed cells 48 h post infection. Antibodies directed against Pmp21 (green) detect chlamydial particles in the inclusion, which were counterstained with DAPI. Note the dotted ring-shaped patterns often seen upon labelling of Pmp21 (marked by white arrows). Bar 1 µm (see also Fig. S3).

Thus far our data indicate that Pmp21-PD on yeast cells or latex beads can bind human cells. Next we analysed the localization of Pmp21 on infectious EBs. Anti-Pmp21-D antibody specifically detects a 55–60 kDa protein in lysates of HEp-2 cells infected with *C. pneumoniae* prepared 36–96 h post infection (p.i.). This probably corresponds to the processed M-Pmp21 identified previously in RBs (Fig. 4A) (Wehrl *et al*., 2004). The high levels found at 96 h p.i. suggested that this form might be present in EBs and, indeed, a strong M-Pmp21 signal was observed in extracts of EBs (Fig. 4B). In EB extracts containing Sarkosyl and DTT, additional weak bands were detected: one of > 170 kDa corresponding to the full-length N/M/C-Pmp21 and one of > 130 kDa – probably N/M-Pmp21 (Fig. 4B). This pattern has been described for Pmp21 isolated 4 days p.i. (Wehrl *et al*., 2004).

Indirect immunofluorescence microscopy of infected cells revealed that Pmp21 can be detected in the chlamydial inclusion and colocalizes with the bacteria at all times analysed. As the *C. pneumoniae* inclusions are completely filled with bacteria we could not inspect the inclusion lumen for possible Pmp21 signals not associated with bacteria. This had been reported for the *C. trachomatis* homologue PmpD (Kiselev *et al*., 2009; Swanson *et al*., 2009). The Pmp21 staining pattern did not change significantly over the course of the infection. Pmp21 was never found outside the inclusion (Fig. S3A). Next we analysed the localization of Pmp21 in greater detail. HEp-2 cells infected with *C. pneumoniae* were fixed with methanol or formaldehyde 48 h p.i., and incubated with the anti-Pmp21-E and an antibody against either the intrachlamydial DnaK or the extrachlamydial OmpA. In methanol-fixed cells, all antibodies reacted with the bacteria in the inclusion. Anti-OmpA antibody, which stains the chlamydial surface, colocalized with anti-Pmp21, giving a ring-like signal (Fig. 4D). In formaldehyde-fixed cells intracytoplasmic proteins are inaccessible to antibodies (the anti-DnaK antibody gave only a very weak signal), while both the anti-OmpA and anti-Pmp21 antibodies reacted, indicating that their targets are located on the bacterial surface (Fig. 4C, PFA; Fig. S3C). At higher magnification in formaldehyde-fixed cells, a dotted ring-like pattern surrounding the DAPI signals representing the bacteria was generated by the anti-Pmp21 antibodies (Fig. 4E; white arrows mark single Pmp21 signals). Microimmunofluorescence of viable, unfixed *C. pneumoniae* EBs confirmed that OmpA and Pmp21 were localized on the bacterial cell surface, while DnaK was only rarely accessible (Fig. S3B).

### Pmp21 is required for infection of HEp-2 cells by *C. pneumoniae*

**Fig. 5:**
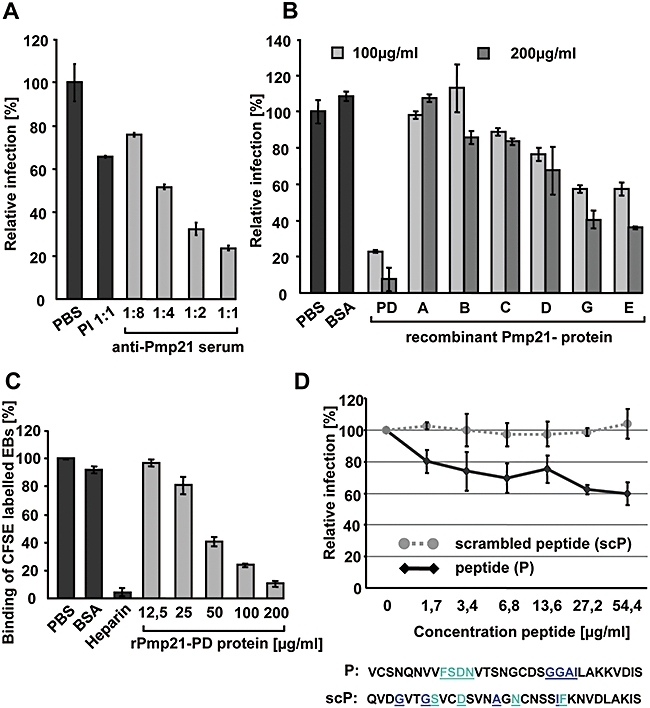
Recombinant Pmp21 inhibits infection by *C. pneumoniae*. Infection of HEp-2 cells by *C. pneumoniae* is inhibited by prior addition of anti-Pmp21, recombinant Pmp21 or a synthetic peptide derived from Pmp21.A. Purified EBs were incubated with PBS, pre-immune serum (diluted 1:1 with PBS) or serially diluted anti-Pmp21-E before being used for infection of HEp-2 cells. The number of inclusions formed was determined by microscopy 48 h p.i. The data in (A), (B) and (D) are derived from four independent experiments for each condition, each involving observation of 20 microscope fields.B. HEp-2 cells (1 × 10^6^) were incubated with PBS, 200 µg ml^−1^ BSA, or 100 µg ml^−1^ or 200 µg ml^−1^ recombinant Pmp21-PD or one of the other Pmp21 variants as indicated prior to incubation with purified *C. pneumoniae* EBs. Cells were fixed 48 h p.i. and the number of inclusions determined as explained above. PBS control = 100%.C. Adhesion of viable, CFSE-stained *C. pneumoniae* EBs to human HEp-2 cells was detected by flow cytometry. Attachment of EBs in the presence of PBS or 200 µg ml^−1^ BSA served as the control, and the fluorescence intensity associated with HEp-2 cells pre-incubated in PBS was set to 100%. Binding of EBs in the presence of 500 U ml^−1^ heparin or increasing amounts of recombinant Pmp21-PD were analysed accordingly. *n* = 2 wells; number of experiments = 6. Error bars indicate standard deviations.D. A Pmp21-derived peptide attenuates *C. pneumoniae* infection. Prior to incubation with purified *C. pneumoniae* EBs, HEp-2 cells (1 × 10^6^) were incubated without or with increasing amounts (1.7–54.4 µg ml^−1^) of a 32-amino-acid peptide derived from Pmp21 (residues 745–776) (peptide) or a scrambled version of the sequence with the same amino acid composition (scrambled peptide). Cells were fixed 48 h p.i. and the number of inclusions determined by microscopy as detailed above. PBS control = 100%.

We then tested directly whether Pmp21 on the EB surface is needed for chlamydial attachment to host cells. Incubation of purified EBs with anti-Pmp21 reduced infectivity of *C. pneumoniae* by up to 80%; while incubation with pre-immune serum only reduced infection by 35% (Fig. 5A). We then blocked Pmp21 binding sites on human cells by pre-incubation of HEp-2 cells with Pmp21-PD and again found strong (∼90%) inhibition of infection by *C. pneumoniae* EBs (Fig. 5B). Pre-incubation with the individual Pmp21 domains shown to bind to HEp-2 cells also reduced infection rates. Pmp21-E and Pmp21-G, which resemble the naturally occurring processed forms N-Pmp21 and M-Pmp21 (see Fig. 1B and C), inhibited subsequent infection by ∼40% (Fig. 5B). Furthermore, binding of infectious EBs stained with the fluorescent dye CFSE to HEp-2 cells was dose-dependently reduced by up to 90% following pre-incubation with Pmp21-PD (Fig. 5C). Taken together, these results demonstrate that EB-associated Pmp21 mediates adhesion of EBs to human cells and is involved in the infection process.

### A synthetic peptide derived from Pmp21 attenuates a *C. pneumoniae* infection

Finally, we asked if a peptide containing a typical tetrapeptide motif doublet derived from Pmp21 could block EB binding and reduce infectivity. We chose the sequence between positions 745 and 776 in M-Pmp21, which has an N-terminal FSDN and a C-terminal GGAI motif. Pre-incubation of HEp-2 cells with the synthetic peptide inhibited infection by *C. pneumoniae* by up to 40%, while a scrambled peptide had no effect (Fig. 5D). This result underlines the importance of the tetrapeptide motifs for Pmp21 function in *C. pneumoniae* infections.

### Pmp6 and Pmp20 mediate attachment of infectious *C. pneumoniae* EBs to human cells

**Fig. 6:**
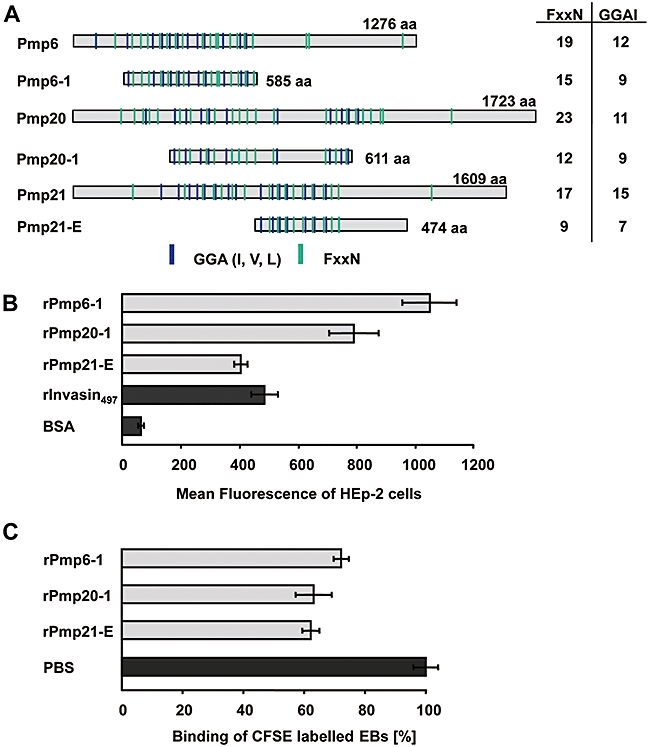
Pmp6, Pmp20 and Pmp21 carry multiple repeats of both tetrapeptide motifs, adhere to HEp-2 cells and are important for infection.A. Schematic representation of the Pmp6, Pmp20 and Pmp21 proteins from *C. pneumoniae*, together with the protein domains analysed here. The position of each of the tetrapeptide motifs GGA(I, L, V) and FxxN is marked and the total number of each motif type found is given on the right.B. Adhesion of Pmp6- or Pmp20-coated fluorescent latex beads to HEp-2 cells. Green fluorescent beads (1 × 10^7^) were coated with (200 µg ml^−1^) BSA, recombinant Inv_497_ (rInv_497_), rPmp6-1, rPmp20-1 or rPmp21-E, and incubated with 1 × 10^6^ HEp-2 cells. Mean fluorescence of HEp-2 cells was determined by flow cytometric analysis. (1 × 10^6^ HEp-2 cells per sample). Data are based on four independent experiments for each protein.C. Adhesion of viable, CFSE-stained *C. pneumoniae* EBs to human HEp-2 cells is reduced in the presence of rPmp6, rPmp20 or rPmp21. The fluorescence intensity associated with attachment of EBs to HEp-2 cells in the presence of PBS served as the control and was set to 100%. Data are based on two independent experiments, each performed in triplicate (*n* = 6). Error bars indicate standard deviations.

Pmp21 is one of 21 Pmps in *C. pneumoniae*. To ask whether other subtypes are relevant for *C. pneumoniae* infections, we chose Pmp6 (subtype B/C) and Pmp20 (G), which resemble Pmp21 (D) in length and in possessing > 30 tetrapeptide motifs in their N-terminal and central regions (Fig. 6A). Fluorescent beads coated with rPmp6-1 or rPmp20-1 adhered more strongly to epithelial cells than beads coated with rPmp21-E or invasin (Fig. 6B). Consistent with this, binding of viable, fluorescently labelled infectious EBs to HEp-2 cells was significantly reduced when the human cells were pre-incubated with 200 µg ml^−1^ rPmp6-1 or rPmp20-1 (Fig. 6C). Recombinant Pmp21-E had a similar effect, confirming the result obtained for the Pmp21 full-length passenger domain Pmp21-PD (see Fig. 5C). Thus, our findings imply that Pmp6, Pmp20 and Pmp21 all contribute to adhesion of *C. pneumoniae* EBs to target cells.

### Pmp6, Pmp20 and Pmp21 are important for infection and exhibit additive effects

**Fig. 7:**
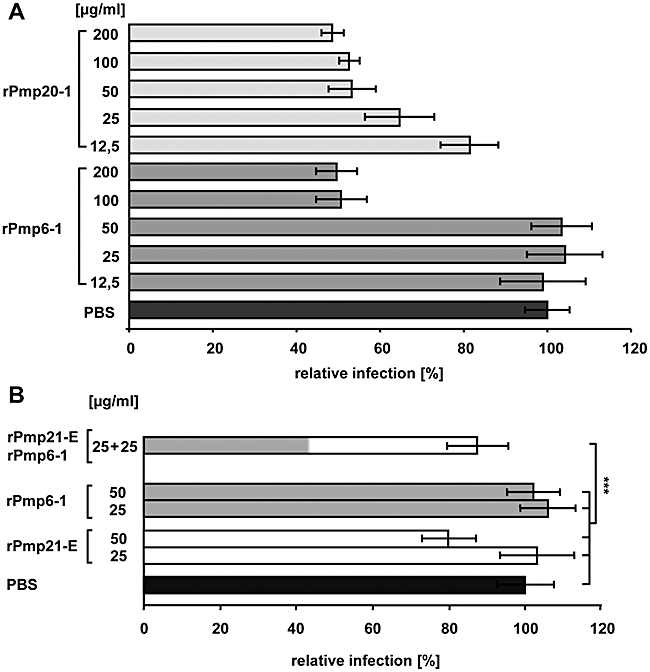
Pmp6, Pmp20 and Pmp21 are important for infection and exhibit additive effects.A. *C. pneumoniae* infection is inhibited by pre-incubation with recombinant Pmp6-1 or Pmp20-1. HEp-2 cells (1 × 10^6^) were incubated with PBS or with different concentrations of recombinant Pmp6-1 or recombinant Pmp20-1 (12.5–200 µg ml^−1^) prior to infection with purified *C. pneumoniae* EBs. Cells were fixed 48 h p.i., and the number of inclusions was determined by microscopy (see legend to Fig. 4 for details). PBS control = 100%. Number of experiments = 4. Error bars indicate standard deviations.B. The infection inhibition assay described in (A) was repeated using a mixture of recombinant Pmp6-1 and rPmp21-E. HEp-2 cells (1 × 10^6^) were incubated prior to infection with purified *C. pneumoniae* EBs with 25 µg ml^−1^ or 50 µg ml^−1^ rPmp6-1 or rPmp21-E alone or a mixture comprising 25 µg ml^−1^ each of rPmp21-E and rPmp6-1. The experiment was otherwise performed as described in (A). PBS control = 100%. ***P-value of 0.001.

Thus far the results obtained for rPmp6 and rPmp20 parallel those obtained for Pmp21. We therefore tested whether Pmp6 or Pmp20 are also relevant for the chlamydial infection. Pre-incubation of HEp-2 cells with recombinant Pmp6 or Pmp20 inhibited subsequent infection by the bacteria in a dose-dependent manner (Fig. 7A). The kinetics of Pmp20- and Pmp21-induced inhibition of infection are very similar, but differ from the results for Pmp6. Nevertheless, these data show that Pmp6 and Pmp20 contribute to EB adhesion and to subsequent *C. pneumoniae* infection.

The data obtained for the three Pmp proteins suggest that the proteins might have overlapping functions. We therefore combined two different Pmp proteins in a single infection blockage experiment. Human cells were pre-incubated with either rPmp6-1 or rPmp21-E protein alone, or a combination of both. Pre-treatment of human cells with 25 µg ml^−1^ rPmp6-1 or rPmp21-E did not reduce infection (106% and 103% of the control values respectively). Interestingly, the combination of 25 µg ml^−1^ rPmp21-E and 25 µg ml^−1^ rPmp6-1 significantly reduced subsequent infection to 87% (*P*-value: 0.001), and 50 µg ml^−1^ rPmp21-E reduced it further (80%), while 50 µg ml^−1^ rPmp6-1 had no effect (102%). These data suggest that soluble rPmp6-1 and rPmp21-E additively inhibit infection, suggesting some overlap in the functions of the native proteins during a chlamydial infection.

## Discussion

Successful infection of host cells depends on a battery of virulence factors, including specialized bacterial surface structures which mediate uptake by host cells (Carbonnelle *et al*., 2009; Kline *et al*., 2009). *Chlamydiae* enter cells via multiple routes, using poorly understood mechanisms (Campbell and Kuo, 2006). In this study we show that Pmp6, Pmp20 and Pmp21 act as adhesins during infection of human cells by *C. pneumoniae*.

Pmps exhibit characteristic features of autotransporters, including proteolytic processing (Grimwood and Stephens, 1999; Henderson and Lam, 2001). Pmp21, like its *C. trachomatis* homologue PmpD, is processed during infection, and processed forms of Pmp6 and Pmp20 have been identified in purified EBs (Vandahl *et al*., 2002; Wehrl *et al*., 2004; Kiselev *et al*., 2009; Swanson *et al*., 2009). Full-length Pmp21 (N/M/C-Pmp21), the intermediate N/M-Pmp21 and the fully processed N-Pmp21 were detected during infection and in infectious EBs, while proteome studies of EBs identified the putative end-products of processing, N-Pmp21, M-Pmp21 and the translocator unit C-Pmp21 (Fig. 1B) (Vandahl *et al*., 2002; Wehrl *et al*., 2004). Using antibodies against the C-terminal part of the PD we detected a protein corresponding in size to the fully processed M-Pmp21 both during infection of HEp-2 cells and on EBs (Fig. 4). Indeed, it appears that the majority of Pmp21 is fully processed during infection, resulting in N-Pmp21 (Wehrl *et al*., 2004), M-Pmp21 (this work) and presumably C-Pmp21 as well. N- and M-Pmp21 remain associated with the bacterial cell surface throughout infection (Fig. 4; Fig. S3) (Wehrl *et al*., 2004). This differs from the more complex set of processed forms described for the homologous PmpD protein from *C. trachomatis* (Kiselev *et al*., 2009; Swanson *et al*., 2009).

Our data show that M-Pmp21 colocalizes with the outer membrane protein OmpA (MOMP), confirming other Pmp21 localization studies (Vandahl *et al*., 2002; Wehrl *et al*., 2004). Interestingly, our anti-Pmp21-E antibody detected dotted, ring-like structures associated with the bacterial cell surface (Fig. 4E). It remains to be seen whether these dots correspond to the oligomeric structures observed for PmpD (Swanson *et al*., 2009). Pmp6 and Pmp20 are also found associated with the bacterial cell surface (Montigiani *et al*., 2002; Vandahl *et al*., 2002). As with Pmp21, the adhesive and infection-relevant properties of Pmp6 and Pmp20 described here were found to reside in their N-terminal segments.

Our studies show that Pmp21-PD (essentially equivalent to N/M-Pmp21), Pmp21-G and Pmp21-E (similar to N- and M-Pmp21 respectively) retain adhesive capacities for HEp-2 cells similar to those found for Pmp21-PD. Interestingly, even small domains of only 50 amino acids (e.g. Pmp21-BΔ2) display undiminished adhesion. Curiously, the adhesive capacity of Pmp21-E and -G is not significantly enhanced relative to that of the smaller fragments. The presence of multiple binding sites on N-Pmp21 and M-Pmp21 could allow them to engage several receptor molecules, or several binding sites on a single receptor molecule simultaneously. We can exclude polymeric glycosaminoglycan structures on HEp-2 cells as potential Pmp21 receptors, as yeast cells presenting Pmp21-B or -D adhered equally well to GAG-deficient CHO lines and the control line K1 (data not shown). GAGs are required for binding of OmcB adhesin, however (Moelleken and Hegemann, 2008), although it is not clear how the activity of OmcB and the Pmp adhesins is co-ordinated during adhesion.

All Pmp21 domains tested showed significant adhesion to HEp-2 cells, while their ability to inhibit infection varied. Pmp21 variants resembling the natural processed forms were most effective, suggesting that these present optimal binding conformations to the receptor(s). This may also explain why the Pmp21-derived peptide had only moderate infection-blocking activity. The inhibitory effect of soluble recombinant Pmp21 protein was confirmed by the neutralizing activity of anti-Pmp21-E antibodies, in agreement with published data for N-Pmp21 and PmpD from *C. trachomatis* (Wehrl *et al*., 2004; Crane *et al*., 2006). Recombinant Pmp6 and rPmp20 also exhibited adhesion to epithelial cells, reduced binding of infectious EBs to HEp-2 cells, and blocked subsequent infection (Figs 6B and C and 7A). Unlike Pmp6, Pmp20 showed a dose dependence similar to Pmp21. This suggests that binding of human cells might vary with Pmp subtype.

All Pmps have multiple copies of the motifs FxxN and GGA(I, L, V) in their N-terminal segments (Grimwood and Stephens, 1999). We found that adhesion of all Pmp21 domains tested required a minimum of two copies of these motifs [2x FxxN or FxxN plus GGA(I, L, V)]. We did not test whether two copies of the GGA(I, L, V) motif are sufficient for adhesion. The distribution of these motifs in Pmps shows no obvious pattern, but the two types often occur as doublets of the form FxxN-x_5–17_-GGA(I, L, V). Indeed, a small M-Pmp21 fragment with a typical doublet (FxxN-x_8_-GGAI) exhibited adhesion and interfered with infection by *C. pneumoniae* (Fig. 5D). The motifs may be required for maintenance of the Pmp conformation that permits adhesion, or might directly mediate interaction with human receptors, or both. Alternatively, the wide variation in the number and spacing of the two motifs could provide a surface unique for each individual Pmp. Repeats of short sequences have been described for other adhesive autotransporters, but no function has been attributed to them (Girard and Mourez, 2006).

Our data show that Pmp6, Pmp20 and Pmp21, representing three of the six Pmp subtypes (G, B/C and D respectively), exhibit very similar functional properties (adhesion, partial inhibition of EB binding to human cells, blockage of subsequent infection). These observations suggest that they have similar or even overlapping functions. In agreement with this, we found that pre-incubation of HEp-2 cells with two different Pmp proteins (rPmp6-1 and rPmp21-E) in a single experiment significantly reduced infection, under conditions where each protein alone was ineffective (Fig. 7B). These preliminary data are concordant with a hypothetical model, in which Pmp6 and Pmp21 have similar but non-redundant roles during adhesion and invasion, possibly recognizing the same host cell receptor or a group of related receptors. We have preliminary data that Pmp20 also participates in this functional network (data not shown). Pmp6 belongs to subtype G, which comprises 13 members in *C. pneumoniae* (Grimwood and Stephens, 1999). Other Pmps of this subtype might have similar roles in infection, as at least nine are reported to be present on infectious EBs (Vandahl *et al*., 2001; Montigiani *et al*., 2002). Indeed, several, or perhaps all, expressed Pmps might together form a supramolecular complex that is required for efficient adhesion. Anti-Pmp21 does not cross-react with Pmp6 or Pmp20 in Western blots (data not shown); nevertheless, it effectively blocks adhesion of *C. pneumoniae*. If these three Pmps were involved in a multimeric complex on the bacterial cell surface, an antibody against anyone could conceivably block adhesion mediated by the others. Indeed, studies in *C. trachomatis* and *C. psittaci* suggest that Pmp PDs could perform homo- or hetero-oligomeric interactions with other Pmps (Tanzer *et al*., 2001; Swanson *et al*., 2009). Those interactions could be modulated by host cell enzymes, e.g. by protein disulphide isomerase (PDI), a surface-located host protein, which is necessary for EB entry (Abromaitis and Stephens, 2009).

The diversity of the Pmp superfamily may also be attributable to antigenic variation as in the case of the Opa protein family in *Neisseria gonorrhoeae* (Grimwood and Stephens, 1999; Carbonnelle *et al*., 2009). This could compromise the host's ability to mount a potent humoral immune response and thus disable host defence mechanisms. This idea is supported by the important finding that Pmp10 from *C. pneumoniae*, as well as all nine Pmps (including the Pmp21 homologue PmpD) in *C. trachomatis*, are variably expressed in inclusions formed in cultured cells (Pedersen *et al*., 2001; Tan *et al*., 2010). This may explain why sera from *C. trachomatis*-infected patients show significant differences in Pmp-specific antibody profiles (Tan *et al*., 2009). If the expression of Pmps on the EB surface also varies in *C. pneumoniae*, the pool of 21 Pmp proteins could form the basis for broad antigenic diversity, while ensuring that EB adhesion properties remain stable.

Pmp proteins may function in concert with other EB surface proteins. For example, the *C. trachomatis* PmpD seems to be in close contact with serovariable MOMP and LPS, as antibodies against either block neutralization by PmpD antibodies, suggesting a decoy-like immune evasion strategy (Crane *et al*., 2006). Likewise, in *C. pneumoniae* Pmp10 apparently protects the C-terminal part of MOMP from proteolytic cleavage (Juul *et al*., 2007). While for the *C. trachomatis* MOMP unspecific interactions with host cells have been proposed (Su *et al*., 1990), we could not detect adhesion of recombinant *C. pneumoniae* MOMP to HEp-2 cells, possibly due to lack of the post-translational modifications that have been found in native *C. psittaci* and *C. trachomatis* MOMPs (summarized in Campbell and Kuo, 2006).

Safe recombinant vaccines, based on a small number of antigenic proteins, are emerging as the most cost-effective way of combating infectious diseases. Adhesins are interesting vaccine candidates, as some provide protection against infection in animal models (Kline *et al*., 2009). A combination of Pmp proteins could yield a very effective vaccine, and would minimize problems associated with antigenic variation that may be linked with this class of proteins.

## Experimental procedures

### Bacterial strains, yeast strains and culture conditions

*Escherichia coli* strain XL-1 blue (Stratagene) was used for protein expression and plasmid amplification. *C. pneumoniae* GiD was propagated in HEp-2 cells (ATCC No. CCL-23) as described (Jantos *et al*., 1997). Chlamydial EBs were purified by using a 30% gastrographin gradient (Schering). The *Saccharomyces cerevisiae* strain yEG2 (*MAT**a** ura*3-52 *trp*1 *LEU2 his*3Δ200 *pep4*:*HIS3 prb*1Δ.6R *can*1 *GAL1p-AGA1::*pIU211 *leu*2Δ1::pCM149) was used for yeast display experiments: the *GAL1* promoter of the chromosomally integrated *GAL1*p-*AGA1* expression unit in strain EBY100 (Invitrogen) was substituted for the KanMX-*tetO_7_-CYC1*p promoter by PCR-based homologous recombination using plasmid pAH3. Strain yEG2 was routinely grown in selective synthetic dextrose (SD) medium plus 2% glucose (Sherman, 1991).

### DNA manipulations and plasmid construction

Plasmids were generated in *S. cerevisiae* as described (Moelleken and Hegemann, 2008). The yeast expression vector pEG2 (this work) was generated by replacing the *GAL1* promoter in pYD1 (Invitrogen) with the *tetO_7_-CYC1* promoter amplified by PCR from pCM189 (Gari *et al*., 1997). The *E. coli* expression vector pKM32 (this work) was constructed by integration of a *CEN6 ARS4 URA3* cassette into *Xba*I/*Pvu*II-linearized pQE-31 (Qiagen). The *pmp21* variants were amplified by PCR using *C. pneumoniae* GiD DNA as template, and cloned into *Not*I-digested pEG vector or fused N-terminally to a His_6_-Tag and cloned into the *Sma*I site in pKM32. *Pmp6* or *pmp20* variants and the *Y. pseudotuberculosis* sequence encoding the invasin fragments Inv_197_ (aa 772–969) and Inv_497_ (aa 490–969) subcloned from pRI285 (Dersch and Isberg, 1999) were cloned into pKM32 as described above. Plasmid pYD1-Inv_197_ has been described before (Moelleken and Hegemann, 2008). Site-specific mutagenesis was performed using oligonucleotides. All constructs were sequenced prior to use.

### Protein expression and affinity purification of His_6_-tagged proteins

For expression of Aga2p or Aga2p fusion proteins in yEG2, cells were grown in selective SD medium and harvested in logarithmic phase. Expression and purification of His_6_-tagged fusion proteins was performed under denaturing conditions following the manufacturer's instructions (Qiagen). Proteins were renatured by dialysis against PBS, and detected after SDS-PAGE by Western analysis with an anti-His antibody (Qiagen).

### Immunoblot analysis

SDS-PAGE and immunoblot analysis were performed as described (Moelleken and Hegemann, 2008). For detection of Pmp21_6His_ proteins, monoclonal anti-His antibodies (Qiagen) or a polyclonal anti-Pmp21-E serum (antigen-purified; IgG 87 µg ml^−1^) (1:250 dilution) was used and visualized with AP-conjugated anti-mouse or anti-rabbit antibodies (Promega). For time-course experiments HEp-2 cells were infected with *C. pneumoniae* at moi 1. After 12–96 h cells were prepared and analysed by SDS-PAGE using a monoclonal anti-β-actin antibody or anti-Pmp21-E serum (antigen-purified). EBs were gradient-purified after 72 h p.i., and 1 × 10^7^ EBs were resuspended in PBS, or in PBS with 2% Sarcosyl, 2% SDS, 10 mM DTT, and sonicated for 1 min at room temperature (RT). Loading dye and 1 mM DTT were added and samples were boiled for 10 min at 100°C. Prior to loading, samples were pelleted for 1 min at 13 000 r.p.m. and supernatant was loaded. Proteins were detected by the anti-Pmp21-E serum (antigen-purified).

### Immunofluorescence microscopy

Twenty-four to 90 h p.i. HEp-2 cells on glass coverslips (12 mm diameter) were washed once with PBS, fixed with freshly prepared 3% formaldehyde (diluted from a 30% formaldehyde stock solution, which was prepared by dissolving paraformaldehyde powder in PBS at 65°C) for 30 min at RT and permeabilized with PBS containing 0.2% Triton X-100 or fixed with 96% methanol for 10 min at RT (Vandahl *et al*., 2002). The monolayer was washed three times with PBS. For detection of chlamydial inclusions or non-fixed *C. pneumoniae* EBs, either anti-DnaK (Birkelund *et al*., 1990), anti-OmpA (Dako) or anti-Pmp21-E serum (antigen purified; IgG 87 µg ml^−1^) (undiluted) was used in combination with Cy3-conjugated anti-mouse antibody (Sigma) or FITC-conjugated anti-rabbit antibody (Dako). For direct staining of inclusions, a monoclonal FITC-conjugated antibody against chlamydial LPS was used (Bio-Rad). Cells were viewed using a Zeiss Axioskop. The microimmunofluorescence (MIF) analysis was performed as described (Moelleken and Hegemann, 2008).

### Yeast cell staining

Approximately 5 × 10^6^ yeast cells from 24 h cultures were washed twice with PBS and immobilized on poly-l-lysine-coated glass slides. Slides were incubated with PBS containing 1% BSA followed by incubation with anti-V5 and Cy3-conjugated anti-mouse antibodies (Invitrogen, Sigma).

### Adhesion assays

Yeast adhesion assays were performed with HEp-2 cells as previously described (Moelleken and Hegemann, 2008). Adhesion assays with protein-coated latex beads were performed as described (Dersch and Isberg, 1999). Alternatively, bead binding to HEp-2 cells was quantified by flow cytometry. HEp-2 cells were grown to a confluent monolayer in 24-well plates and incubated with a 10-fold excess of protein-coated latex beads (diameter 1 µm, green fluorescent, Sigma) for 1 h at 37°C. Cells were washed twice with PBS, detached with Cell Dissociation solution (Sigma) and analysed by flow cytometry using a FACSAria (BD Biosciences).

### Infection inhibition assays

These assays were performed as previously described (Moelleken and Hegemann, 2008). The anti-Pmp21-E serum (total protein content 3 mg ml^−1^) was used without further purification as outlined in the legend of Fig. 5A. Statistical analysis of the infection inhibition assay was carried out by Student's *t*-test.

### Flow cytometric assay for adhesion of CFSE-labelled *C. pneumoniae* EBs

Approximately 2 × 10^8^ purified *C. pneumoniae* EBs were labelled for 1 h at 37°C with 25 µmol of CFSE (Molecular Probes) and washed twice with PBS containing 1% BSA as previously described (Schnitger *et al*., 2007). Confluent monolayers of HEp-2 cells grown in 24-well plates were incubated for 4 h at 37°C with 12.5–200 µg ml^−1^ recombinant protein or 500 µg ml^−1^ heparin, CFSE-labelled *C. pneumoniae* EBs (moi 10) were added for 1 h. Cells were then washed with PBS, trypsinized and fixed with formaldehyde, and the efficiency of adhesion was measured by flow cytometry using a FACSAria (BD Biosciences).
