# Supplementary material for: Members of the Pmp protein family of Chlamydia pneumoniae mediate adhesion to human cells via short repetitive peptide motifs
Source: Mol Microbiol. 2010 Oct 6;78(4):1004–17. doi: 10.1111/j.1365-2958.2010.07386.x (PMC2997323; doi:10.1111/j.1365-2958.2010.07386.x)
Supplement: Supplementary file 1 [file mmi0078-1004-SD1.pdf]

Supplementary Material

Members of the Pmp protein family of *Chlamydia pneumoniae* mediate adhesion to human cells via short repetitive peptide motifs

Katja Mölleken,*, Eleni Schmidt * and Johannes H. Hegemann [#]

*** Alphabetical order; the first two authors contributed equally to this work.**

[#] Corresponding author: Johannes H. Hegemann;
Institut für Funktionelle Genomforschung der Mikroorganismen;
Heinrich-Heine-Universität Düsseldorf;
Universitätsstr. 1, Gebäude 25.02.U1;
40225 Düsseldorf; Germany
Tel.: (+49)211-81-13733;
FAX: (+49)211-81-13724;

Email: johannes.hegemann@uni-duesseldorf.de

Supplementary Figure S1: Diagrammatic representation of the yeast display system.
The protein of interest (protein X) flanked by protein tags is presented on the yeast cell surface as a fusion product linked to the yeast surface protein Aga2p. Labeling with antibodies directed against Pmp21 reveals that the fusion protein (red) is present on the cell surface in living, unfixed yeast cells which express GFP (green), as confirmed by the merged image on the right. Bar 1 µm.

Supplementary Figure S2: *C. pneumoniae* OmpA shows no adhesion in the yeast adhesion and the bead assays.
Yeast cells expressing Aga2 or Aga2-Inv_{197} or Aga2-OmpA were incubated with $1x10^5$ HEp-2 cells, and the number of yeast cells adhering to HEp-2 cells was determined by microscopy. (N = 1000 HEp-2 cells, No. experiments = 4).
Adhesion of latex beads coated with the indicated proteins to HEp-2 cells. Beads (1 x 10^6) coated with 100 µg BSA, rMBP, rMBP-Inv_{197} or rMBP-OmpA were incubated with 1 x 10^5 HEp-2 cells and the number of beads associated with HEp-2 cells was determined by microscopy.
Error bars indicate standard deviations.

Supplementary Figure S3: Pmp21 is expressed during infection and EB cell surface located.
(A) Photomicrographs of HEp-2 cells infected with *C. pneumoniae* at moi 1 in the presence of 1.2 µg ml^{-1} cycloheximide. For a time course of expression of Pmp21 during infection, HEp-2 cells were infected with *C. pneumoniae* strain GiD and were fixed with methanol at the indicated time points, and subjected to immunostaining with anti-OmpA (red) and anti-Pmp21 (green) antibodies. The overlay of OmpA and Pmp21 staining is shown in the rightmost image in each row (merge). Bar 5 µm.
(B) Localization of Pmp21 on purified, non-fixed *C. pneumoniae* EBs. Equal amounts of EBs were used for microimmunofluorescence (MIF) analysis. *C. pneumoniae* antigens DnaK, OmpA and Pmp21 were detected using anti-DnaK, anti-OmpA and anti-Pmp21 antibodies. Bar 1 µm.
(C) Differential fixation for the detection of intra-chlamydial versus extrachlamydial proteins. *C. pneumoniae* infected HEp-2 cells were formaldehyde (PFA) or methanol (methanol) fixed as described in Materials and Methods and stained with the polyclonal antibody against the cytoplasmic ribosomal protein S1 (kindly provided by Birkelund). Formaldehyde-treated cells are not accessible for the antibody, while methanol-fixed cells allow the staining of the ribosomal S1 protein, as described by ((Mygind *et al.*, 1998).

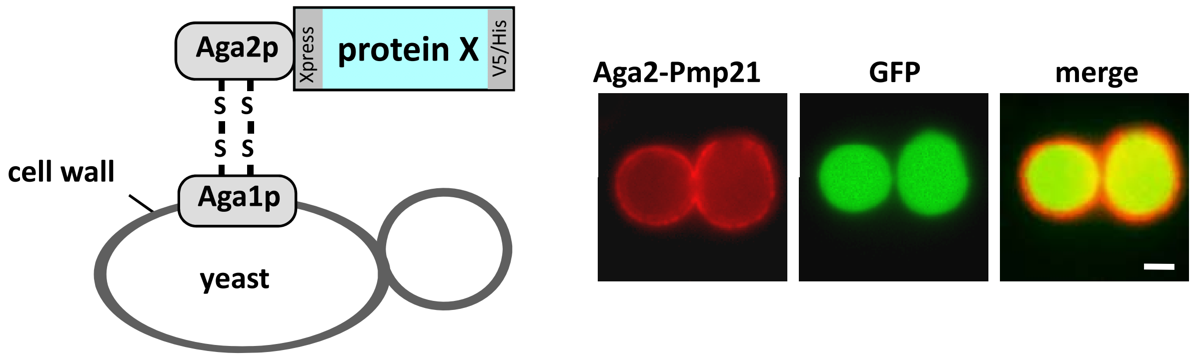

Supplementary Figure S1

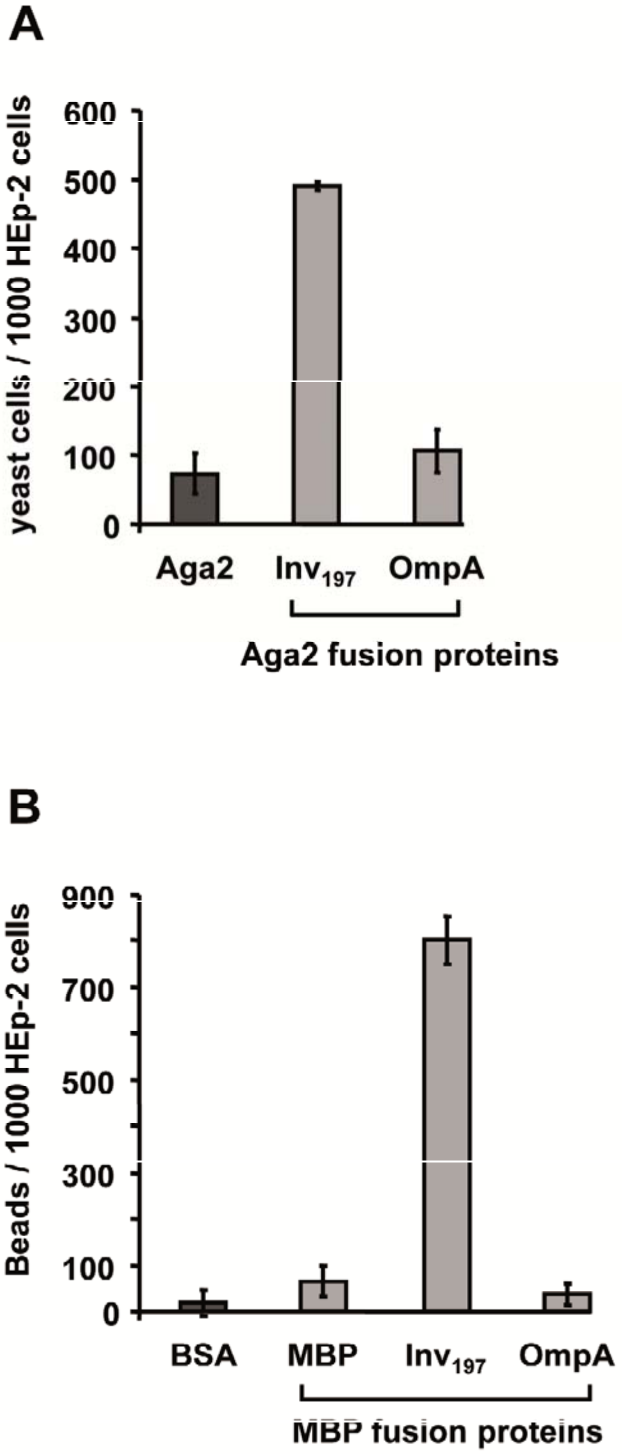

Supplementary Figure S2

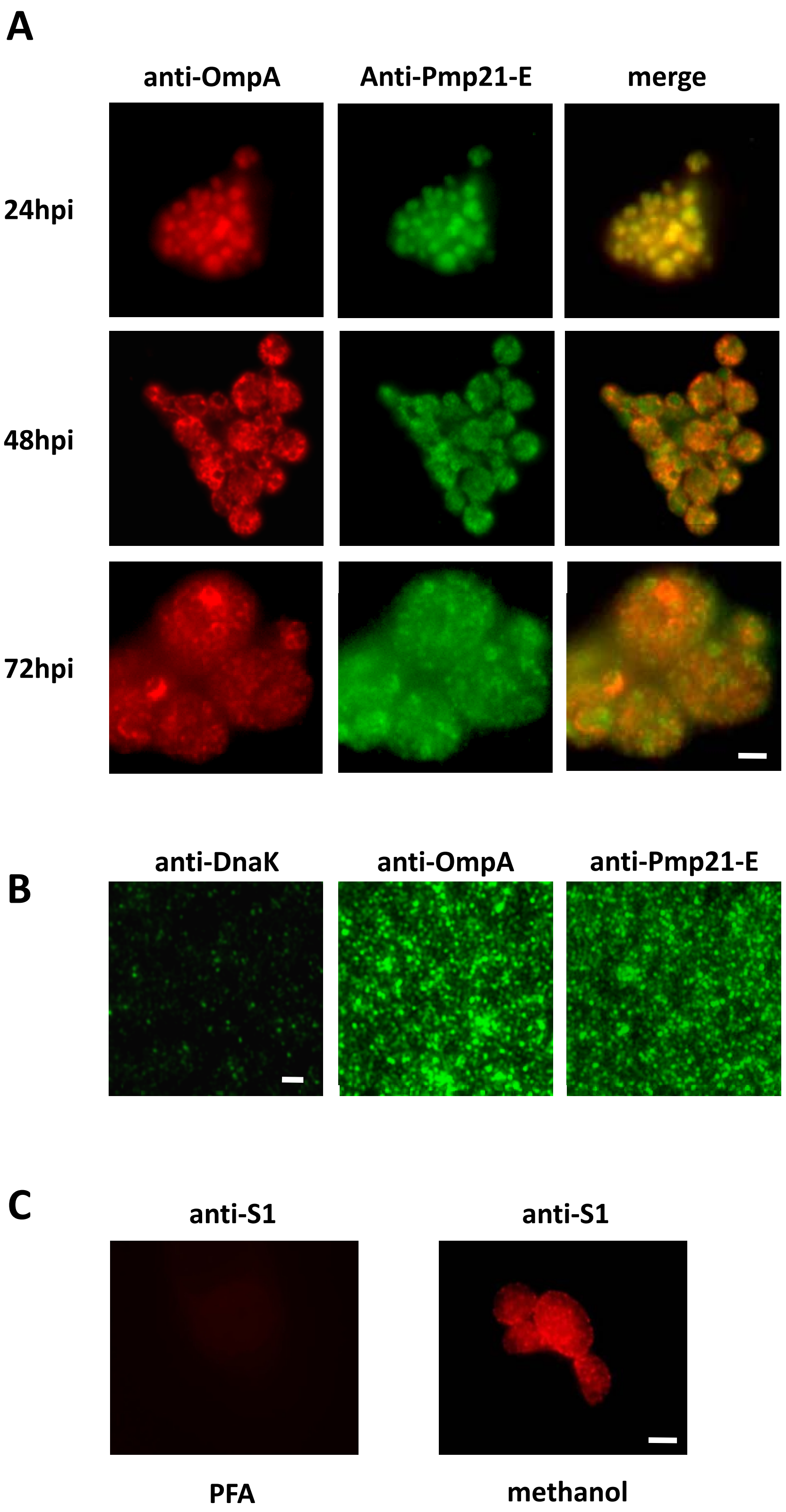

Supplementary Figure S3
